# Research ethics committees in the regulation of clinical research: comparison of Finland to England, Canada, and the United States

**DOI:** 10.1186/s12961-016-0078-3

**Published:** 2016-01-19

**Authors:** Elina Hemminki

**Affiliations:** THL (National Institute for Health and Welfare), P.O. Box 30, 00271 Helsinki, Finland

**Keywords:** Ethics, Institutional review boards, Patient protection, Quality assurance, Research ethics committees, Research policy

## Abstract

**Background:**

The aim of this paper is to compare common features and variation in the work of research ethics committees (RECs) in Finland to three other countries – England, Canada, the United States of America (USA) – in the late 2000s.

**Methods:**

Several approaches and data sources were used, including semi- or unstructured interviews of experts, documents, previous reports, presentations in meetings and observations. A theoretical framework was created and data from various sources synthesized.

**Results:**

In Finland, RECs were regulated by a medical research law, whereas in the other countries many related laws and rules guided RECs; drug trials had specific additional rules. In England and the USA, there was a REC control body. In all countries, members were voluntary and included lay-persons, and payment arrangements varied. Patient protection was the main ethics criteria, but other criteria (research advancement, availability of results, payments, detailed fulfilment of legislation) varied. In all countries, RECs had been given administrative duties. Variations by country included the mandate, practical arrangements, handling of multi-site research, explicitness of proportionate handlings, judging scientific quality, time-limits for decisions, following of projects, role in institute protection, handling conflicts of interests, handling of projects without informed consent, and quality assurance research. The division of work between REC members and secretariats varied in checking of formalities. In England, quality assurance of REC work was thorough, fairly thorough in the USA, and not performed in Finland.

**Conclusions:**

The work of RECs in the four countries varied notably. Various deficiencies in the system require action, for which international comparison can provide useful insights.

**Electronic supplementary material:**

The online version of this article (doi:10.1186/s12961-016-0078-3) contains supplementary material, which is available to authorized users.

## Background

Review by research ethics committees (RECs), also called research ethics boards or institutional review boards, has become the key in clinical research regulation [1-4]. In the European Union (EU), the 2001 Clinical Trial Directive, which required the establishment of ethics committees to review drug trials, was an important stimulus for the appearance of such bodies. As part of a larger study on clinical research regulation, the work of RECs in Finland was compared to three other countries: England, Canada, and the United States of America (USA) [4]. This paper aims to compare common features and variation in the work of RECs in the late 2000s, concentrating on the main REC system. The comparison of REC structures and the study context have been previously published [4], and are only briefly summarized below.

There is much previous literature on the work of RECs in individual countries and their specific aspects; anecdotal stories and expert views have also been published. Comparative data on decisions and the quality of REC work are scarce, but studies show that countries vary in terms of the structural features of their RECs [1,5-14].

In Finland, RECs are area-based and have a monopoly, handling all medical research (as defined by law) in their area. Only a small and regulated number of RECs deal with clinical research (21 (five since 2010) and a central committee); there are no private RECs. In England, since 2004, the number of RECs has been regulated, declining from around 200 in 2002 to around 80 in 2010 (69 in 2013). England’s few private RECs were amalgamated with the NHS system by 2013. In Canada, RECs are health facility (hospital)-based, substance-based, or private, can be freely established, and are numerous. Likewise, the USA has a large number of RECs (around 4,500), and several RECs can be located within one institute or hospital. Private committees play a bigger role, being large in size but small in number (around 30). All four countries have RECs other than those described above, which handle health research beyond the mandate of the main REC system.

## Methods

The methods used herein have been described in detail in previous papers [4,15,16], and briefly summarized below. Several approaches and data sources were used. The key method applied consisted of semi-structured and unstructured interviews of experts. In addition, use was made of previous reports, documents, meeting presentations, informal discussions with experts and observations. Empirical data was systematically collected: in Finland in 2009–2011, in Canada in late 2010, in England in early 2011, and in the USA in late 2011. Some data was completed up to spring 2014 using web pages, publications and later interviews.

In Finland, 26 experts involved in research and health services were purposefully selected and interviewed, as were 22 chairpersons from 25 official medical RECs. In the other countries, interviewees were chosen based on previous knowledge of institutes important in this context, suggestions made by the interviewees, geographical proximity, and availability during data collection visits. The numbers of interviewed experts were: 21 in England, 13 in Canada (Ontario), and 24 in the USA. In spring 2014, a draft report was shown to two experts in Canada, England, and the USA and was modified based on their comments.

Most interviews were semi-structured, but some were unstructured and resembled normal discussions. The themes of the interviews and some pre-prepared questions were drawn up prior to each interview, but the actual interview and its focus varied in accordance with the expert’s position, experience and emergent information. Information and material from previous interviews were utilized in subsequent interviews. In Finland, the original questions and themes were chosen by previous literature and the project researchers’ knowledge and experience of research regulation; the questions were reformulated when new information from the interviews accumulated. In the other countries, the interviewees were approached with an open mind. Data collection was a learning process: the questions and items were first formulated by the Finnish experience and prior reading, and reformulated in subsequent interviews by new information.

The interviews lasted from 30 minutes to 3 hours. They were not tape-recorded, but notes were made and a summary of the interview was drawn up afterwards. In each country, documents were collected from the web pages of the institutions involved, or were handed over during the interviews. The relevant publications were sought from literature databases and from references given during the interviews.

For the analysis, a theoretical framework was created comprising the dimensions of REC structures and work. The analysis was material based (grounded theory). During data collection, preliminary classes of items were raised and noted. After the data were collected, empty tables, organized by dimension each containing many items, were created. The tables were initially filled, as recalled from the data collection. Then, country-specific interview notes and documents were iteratively read by one researcher; notes were made on various topics and dimensions using self-adhesive notes, and organized by topic/dimension and by country. The framework was modified and made more detailed during note making. Data from various sources was synthesized and the features of the systems were simplified and classified. If an item was an unequivocal fact and not found in the original country-specific notes, documents and web-pages were searched for it.

Different terms, varying between the interviewees and documents, were used in each country. Specific names and terms were changed into general ones [4]. Clinical research refers to research conducted on and with patients; clinical trial refers to research that evaluates interventions using experimental methods (not restricted to drugs).

A positive statement was issued by the THL ethics committee on the project as a whole (MERGO Ethical review and administrative governance of clinical research; June 17, 2010, amendment Jan 27, 2011). All interviews were voluntary; the interviewees understood the purpose of the interviews and were interviewed as experts. The documents used were public.

## Results

### Legal and ethical basis

In Finland, the law-based RECs held a monopoly on reviewing all “medical research” (Table 1), but could review other types of research by choice. The definition of medical research was narrow: “research, in which the integrity of a human or her embryo or fetus is subject to intervention or the causes, symptoms, diagnostics, care, prevention or general features of diseases, (since 2010) and which aims to increase knowledge on health”, leaving out much health and healthcare research. Until 2008, in England, all research within the NHS, all research with NHS patients since then, and some specific types of research defined by various laws, were to be handled by NHS RECs. In Canada and the USA, RECs covered human research broadly, but different types of research projects were often handled by different RECs. The definition of human research was restricted to identifiable private information and living humans.

**Table 1:** Rules regulating research ethics committees (RECs) and their work in clinical research, Finland compared to England, Canada (Ontario), and the USA, around 2010

	Finland	England	Canada	USA
Type of research handled	Medical	Within NHS; specific types^a^	Human and health care	Human and health care
Specific research law	Yes, medical research	No	No	Yes, general
Main basis	Law	Health Ministry rules	Requirements from national grant agency	Government rules
Special law for drug trials	EU clinical trials directive, national laws	EU clinical trials directive, national laws	Drug and devices law	Drug, devices and food law
Leading document	No single	No single	Tri-Council statement^b^	Common rule
Helsinki declaration	Separate document	Separate document	Some parts integrated in leading document	Some parts integrated in leading document
International Committee on Harmonization (drug trials) Good Clinical Practice guidelines	Separate document for drug trials	Integrated to rules	Integrated in leading document	Integrated in leading document

^a^Regardless of place, a certain type of research, such as investigational drugs and devices, adults lacking capacity to consent, exposure to ionizing radiation.

^b^Tri-Council statements, tri-council policy statement: ethical conduct for research involving humans.

In each country, there were many rules and guidelines, which RECs should or could use to guide their work. These are described in simplified format in Table 1 and Additional file 1. In Finland, RECs had been regulated since 1999 by a medical research law and its sub-laws, specifying REC mandates and tasks. In the other countries, RECs were less directly law based, but referred to by various laws. In England, RECs were specified by health ministry rules, including those of the REC control body (National Research Ethics Service (NRES); in 2012, it became part of the Health Research Authority). Although RECs were not legal bodies in general, they were in regard to drug and device trials and some special classes, such as research with persons lacking the capacity to consent and exposure to ionizing radiation, as stipulated in specific laws. In Canada, RECs were formalized by the requirements and guidelines of the main public funder, the Canadian Institutes of Health Research, part of the Tri-Council funding agency. In the USA, the research law (1974 National Research Act) mentioned the need for REC review and informed consent. REC tasks were expanded in government (federal) regulations (Common rule). The Common rule concerned all human research, but was adaptable to the research type and purpose.

In each country, laws on data confidentiality (privacy laws/freedom of information laws) existed and were integral to research regulation. Detailed information on these intricate regulations was not collected, and the role of the RECs overseeing them varied. Additional special laws governed drug trials in all of the countries [4].

Research ethics codes, such as the Declaration of Helsinki [1], were not central to discussions on the REC system or work. However, codes had had an impact on national guideline formulation.

### REC members and processes

#### Members

In Finland, expertise in research ethics, law, medicine, nursing sciences (since 2010) – and at least two lay-members – were required (Table 2). In England, a varied composition was required, with one third of the members being lay-persons. In Canada, the required expertise was defined as multidisciplinary and independent: at least five members, both men and women, of whom at least two were experts in the research area, one in ethics, one in the relevant law, and one community representative. In the USA, the regulations defined the minimum number of members (five), at least one of whom was to be unaffiliated with the institution. Membership should be diverse (by race, gender, cultural background, sensitivity to community issues), with at least one scientist and one non-scientist. In all countries, REC membership was voluntary.

**Table 2:** Members and processes of research ethics committees (RECs), Finland compared to England, Canada (Ontario), and the USA, around 2010

	Finland	England	Canada	USA^a^
REC members				
Expertise	Specified some expertise	“Many-sided”	Specified some expertise	“Many-sided”
Lay-members, at least	Two	One third	One	One
Voluntary	Yes	Yes	Yes	Yes
Payment	Varied, remunerations	No, locum costs covered	Varied, chairpersons usually paid	Varied, members usually modestly
Process				
Multi-centre project	One committee	One committee	Usually several	Various options, usually several
Time-limit for decisions	3 months for drug trials	2 months for drug trials	No	No
Proportionate review	Not explicitly	Yes, leading principle	Yes	Yes, leading principle
Help researchers in submission	Most RECs	Yes	Yes	Yes
Application formalities	Also members	Secretariat mainly	Secretariat mainly	Secretariat mainly

^a^Private RECs different.

Payment arrangements varied by REC and country; furthermore, the employers of REC members decided on whether work could be done during working hours. In Finland, meeting costs were usually covered (Table 2). In some committees, the chairperson and the member presenting the application were paid a lump sum. In England, members and chairpersons were unpaid, but locum (cover for members during REC meetings) and travel costs could be covered. In Canada, REC chairpersons were usually paid, but members were not. Some REC chairs were professional and paid for their weekly time. In the USA, institutional members could count REC work as part of their share of outside funding. Private committee members were paid.

#### Multi-centre project handling

In Finland, the research law’s amendment in 2010 was explicit to only a single handling. The central REC was the primary address for multi-centre drug trials, but most applications were delegated to local RECs. In England, the EU clinical trials directive contributed to the creation of a system in which multi-centre projects were reviewed by one REC only. In Canada (Ontario), several proposals had been made to centralize REC work for multi-centre projects, but this had occurred in only a few areas, such as cancer trials. In the USA, multi-centre projects could be handled in three ways: in each local committee, one committee being chosen to review on behalf of all, or an outsider ethics committee being chosen to conduct a single review. Even though the problems associated with multi-handling were obvious, various factors favoured decentralized handling. They included close collaboration between RECs, the decision maker and the research contract office, liability issues and hospital insurers’ requirements.

#### Time-limits for decisions

In Finland and England, the EU clinical trials directive set limits on review times and tabling for drug trials (Table 2), but did not apply to other types of research. In Canada and the USA, there were no rules on how quickly a REC should make decisions or the number of times it could table an application. In all countries, researchers and sponsors expected rapid handling and clear reasons for tabling; anecdotal information suggested that many researchers were dissatisfied with these aspects.

#### Presence of researchers

In Finland, researchers were seldom present in REC meetings to answer questions, in England this was standard practice, but in Canada (Ontario) it was rare. No information was available for the USA.

#### Proportionate review (different handling based on assumed risk to study participants)

In Finland, proportionate review was not explicit (Table 2). In England, proportionate review was pushed by higher level research regulators. In Canada and the USA (the terms ‘expedited review’ and ‘fast tracking’ were used) proportionate review was explicit. The division into ordinary and expedited reviews was usually proposed by the researchers and preliminarily determined by the REC secretariat.

#### Checking formalities

In all countries, REC secretariats often helped researchers to prepare the application. However, in Finland, the secretariats were modest in size and technical capacity, reducing the help they could offer (Table 2). REC secretariats were mainly responsible for ensuring that the applications fulfilled the various detailed rules, but REC members also performed much of this work. Among REC members, a common complaint was that ethics are buried under technical details. In England, the REC control body (NRES) had successfully lightened the administrative burden, including the introduction of a standard electronic portal for applications. In Canada, technical assistants for paperwork were commonly organized into ethics and contract offices. Applications were constructed based on standard forms, but REC members and researchers still complained about spending too much time following rules. In the USA, the application forms were detailed and institutions had created various well-resourced offices to lessen the REC’s work.

#### Costs

Institutions’ regulatory costs associated with RECs were mentioned in expert interviews only in the USA, being considered an important risk management element. Regulatory costs for researchers, including monitoring costs, emerged as an issue in Canada and the USA. The requirements set by RECs and other research regulators could consume a large part of the budget. In Canada, one interviewee considered high costs an unfair inhibition on researchers with small budgets.

### REC tasks

#### Patient protection

In all countries, the starting point for REC work was patient protection (safety), including voluntary participation (patient autonomy) and data protection (confidentiality) (Table 3). In the absence of specific criteria, the way in which patient protection was measured and balanced with other study aspects varied.

**Table 3:** Tasks and items in clinical research projects covered by research ethics committees (RECs), Finland compared to England, Canada (Ontario), and the USA, around 2010

	Finland	England	Canada	USA
Patient protection	Starting point	Starting point	Starting point	Starting point
Research advancement	No	Yes	Yes	Yes^b^
Research prioritization	No	No	No	No
Resource^a^ competition	No	No	No	No
Follow-up	No	Yes	Yes	Yes
Contract	No	No, another body	No, another body	No, another body
Items				
Scientific quality	Yes	Evidence asked for	Yes/ no	Yes/Evidence asked for
Availability of results	No	Yes	Yes	Yes^b^
Research registration	No	Yes	Yes (trials)	Yes (trials)
Institutional liability	No	No	Important	Important
Following (legal) rules	Important	Not major issue	Unknown	Unknown
Conflicts of interest	No	Yes	No, another body	No, another body

^a^Patients, doctors and other resources for research.

^b^Unsure.

#### Research advancement

In Finland, the advancement of (good) research was a concealed task (Table 3), although some REC committees or individual members were aware of it. The benefits of research were usually narrowly defined in relation to the participants in the project under review. In other countries, particularly England, research advancement was more explicitly acknowledged and the benefits for future patients were considered.

#### Research prioritization

Was not considered a REC task in any of the countries (Table 3). Neither were RECs interested in competition for resources (availability of eligible patients and professionals) within an institute or population. This potentially favoured sponsors who could offer better terms to the institute or health professionals conducting research.

#### Responsibility towards researchers

The consequences of REC work for researchers or research funders, in terms of time or resources, were not a REC consideration. An exception was drug trials in Finland and England, for which the EU directive set time limits on decision making (Table 2). Researchers and sponsors had to pay the extra costs or delays resulting from REC requirements. This was particularly problematic in the case of academic funding, which could be small with tight time limits.

#### Follow-up

Unlike the other countries, RECs in Finland were to review the research plan but not follow the project (Table 3). Researchers or sponsors could voluntarily send amendments for review, which commercial sponsors often did. The European clinical trials directive required the follow-up of drug trials, but this was done by the drug authority. In the other countries, RECs had to follow the projects and annual reporting was customary.

#### Contracts

In Finland, RECs did not inspect contracts between sponsors and institutions/researchers, other than the size of intended payments to physicians (Table 3). Research sites (e.g. hospitals) made contracts, giving no feedback to RECs. The same occurred in England, where contracts were made by healthcare trusts, with little cooperation between RECs and trusts. In Canada and the USA, contract offices acted separately from RECs, but informed each other of their evaluations and decisions.

### Specific items

#### Scientific quality

In all countries, the scientific quality of a project was considered an ethical prerequisite: quality is important in evaluating the risk-benefit ratio and poor quality research needlessly puts patients at risk. The role of RECs in this evaluation varied, and there could be parallel reviews, including those of funders (Table 3). In Finland, this evaluation was a REC task, whereas in England RECs had clearly been deemed unsuitable for this role. Instead, researchers or sponsors were asked to provide evidence of scientific quality for REC consideration. In Canada and the USA, reviewing scientific quality was often a REC task. Sometimes, a tension existed between established researchers and REC capabilities: established researchers resented RECs’ involvement in methodological questions. In the USA, the drug control authority thoroughly reviewed the scientific aspects of clinical trials involving new drugs and devices or their new indications.

#### Availability of results

In Finland, RECs did not cover the availability of results (publication) (Table 3), even when it was clear that the researchers had no control over publication. Registration of trials or other research was not required. However, many trials were registered in international registers due either to anticipated publication requirements or the trial being international. In England, the standard nationwide REC application form asked for a publication plan and trial registration. In Finland and England, drug trial registration in an EU register was required by an EU directive.

In Canada, checking on the availability and assumed publication of results was part of REC work. At least in the studied RECs, contract offices checked that no clause prevented publication, which would be grounds for rejecting the study. In the USA, registration of drug and device trials was required by law and presenting the outcomes in a public register had been proposed. For other types of research, registration was voluntary.

#### Legality

This covers two related aspects: the role that RECs had played (1) in ensuring that laws and other rules were followed and (2) in protecting institutes and researchers from liability (Table 3). In Finland, checking that the project abided by laws and other rules was a key task. RECs had a small role with regard to liability. In England, a REC could define a project as ethically sound even when it broke a law or other rule. However, permission would scarcely be forthcoming from the institute for such a project.

In Canada, the Tri-Council statement offered ambivalent advice on how RECs should take account of laws and rules, but each REC was required to have a person familiar with the relevant laws. In the RECs on which information was available, the assessment of ethics and legality were separated in the evaluation. Insurance companies’ requirements and other factors suggested that institutional liability was a consideration. In the USA, although the REC secretariat checked the legal requirements, RECs also devoted time to legal issues. RECs played a major role in protecting institutions. If approval of a study later turned out to be wrong, the entire institute’s research could be endangered. Investment in RECs was a key risk avoidance method within institutions.

#### Conflicts of interest (COI)

In Finland, COI between the researcher/institute and sponsor were not part of ethical judgments (Table 3), and no other body oversaw COI. Since the late 2000s, RECs had been advised to ask about payments to physicians in order to assess the temptation to lock patients into a research project, but no transparency was required towards research participants. COI within RECs were carefully observed, based on the legislation on civil servants. In England COI checking was an REC task, though no information was available on its thoroughness. In Canada and the USA, COIs and their disclosers were important; special offices or bodies took care of them and informed the RECs of their conclusions. In Canada, patient leaflets usually included a description of the financing source.

### Quality assurance of REC work

#### REC control body oversight

In England and the USA, RECs were regulated and supported by a REC control body (Table 4). In Finland, one of the central REC’s (the National Committee on Medical Research Ethics) tasks was to provide local RECs with guidance and education and to give second opinions in the case of complaints, but it had no formal power over local RECs and had not taken a leadership role.

**Table 4:** Features related to quality of research ethics committee (REC) work, Finland compared to England, Canada (Ontario), and the USA, around 2010

	Finland	England	Canada	USA
REC control body oversight	–	Strong	–	Distant
Formal quality assurance	No	Control body	Accreditation planned	Control body, voluntary accreditation
Inspection of RECs	No	Control body, rarely by drug authority	Rarely by drug authority	Control body and drug authority
REC dismissal	Not in practice	Yes by central REC	Not in practice	In theory by central REC
Researchers’ choice of REC	No	Yes	No	Varied^a^
REC decision	Statement	Approval	Approval	Approval
Appeal possibilities	Limited	Yes, clear system	Yes	In theory
Independence from research site	Semi-independent	Yes	No	Varied
Accountable to	Hospital district^b^	Control body	Hospital board^c^	Institution/None
Transparency	Low	High	Low, recognized	Low
Obligatory education of members	No	Yes	No	No
Variability of decisions	Not discussed	Action taken	Identified problem	Identified problem
Dealing with exceptions				
Informed consent exemptions in trials	Not	Yes	Yes	Yes
Emergency drug trials	Not possible	Possible	Possible	Possible
Handling of quality assurance research	Ambiguity	Problem identified	Varied, problem identified	Varied, problem identified

^a^Not in established academic research centres, elsewhere possible.

^b^Since 2010, before then accountability unclear.

^c^Hospital boards consisting of outside members.

In England, the NRES had been active in improving REC work, reducing REC numbers, and streamlining and standardizing procedures. It appointed, credited and audited local RECs and educated their members. REC secretariats were employees of the NRES. The NRES audited RECs through visits, document checking, statistical follow ups, and feedback from researchers. The NRES did not review applications. In case of a complaint, it referred the application to another local REC for a second review.

In the USA, the Office of Human Research Protection (OHRP) registered RECs and issued an “REC license” (a federalwide assurance) by application. It could perform or commission site visits to RECs or research sites. In the case of (suspected) misconduct in a study, the OHRP could overrule the REC decision and all approvals made by the REC. Researchers could complain to the OHRP, but the OHRP did not perform reviews itself. It was more distant than the NRES, having no day to day interaction with local RECs. It had small resources in relation to the number of RECs. The OHRP hosted a national committee (Secretary’s Advisory Committee on Human Subjects Research), which provided expert advice and recommendations on ethics and research regulation.

#### Formal quality assurance

Unlike the other countries, Finland had no formal quality assurance of RECs (Table 4). In England and the USA, quality assurance was performed by the REC control bodies. In addition, the drug authorities could inspect RECs that had handled drug or device trials. In the USA, it could sanction and even disqualify RECs. In the USA, voluntary accreditation of RECs, especially private ones, was common, performed by a private company and costly. In Canada, there were plans to establish an accreditation system, but by 2011 this was not in place for human RECs. However, hospitals were accredited, obliging them to demonstrate that ethics were being attended to. Private RECs were unregulated. The Canadian General Standard Committee (a federal agency) had begun introducing standards for RECs dealing with drug and device trials. The drug authority occasionally inspected RECs performing drug and trial reviews.

#### REC dismissal

In England, the NRES could suspend a REC (Table 4). In the USA, the OHRP could temporarily stop the work of a REC and in theory dismiss it. In Finland and Canada, questions regarding dismissal were not specifically asked, but there were no clear practices for this and, to the author’s knowledge, it had never occurred.

#### Researchers’ choice

In Finland, researchers could not choose the REC, which was determined by the professional location of the principal investigator (Table 4). In England, researchers could choose a REC within the NHS, which then had to handle the application, but the distance travelled for presenting plans and waiting lists for popular RECs regulated client numbers. Besides specific groups defined by law, RECs did not handle research outside the NHS. In Canada, research on patients cared for in a health facility receiving government funding had to be handled by the REC for that facility, unless an agreement existed with a central committee. In the USA, researchers in academic institutions had to use the REC assigned by the institute. Researchers outside academic institutes could choose the REC, but institutional RECs seldom reviewed external research projects.

#### Researcher’s appeal possibilities

In Finland, a positive statement was made rather than a decision (Table 4), although the statement was tantamount to a decision in regard to obtaining research permission. However, since this was not a decision in legal terms, researchers could not complain, but could only request the REC to consult the central REC for a second opinion. There was no right to appeal in the case of projects handled by the central REC itself. Researchers could re-submit to the same committee in the case of a negative decision.

In England, in the case of rejection, the researcher could discuss the issue with the REC, resubmit the application to the same or another committee, or appeal to the control body (NRES). In Canada, the researcher could discuss the issue with the REC. If no agreement was reached, the researcher could appeal to an institutional appeal committee. In the USA, the author had the impression that researchers or sponsors could negotiate with RECs but did not often appeal, although appeals could be made to the OHRP.

#### REC independence from research sites

In Finland, RECs were based in central hospitals. As most clinical research occurred there, RECs were only semi-independent from the research sites (Table 4). After 2010, the number of RECs was reduced and more projects were evaluated outside the responsible institution. In England, RECs were administratively independent from research sites. In Canada and the USA, RECs were mostly within the institute. Private RECs were independent of research sites.

#### Accountability

In Finland, the nomination of REC members and the accountability of RECs were unclear until 2010. Since then, REC members had been appointed by university hospital districts (Table 4). Although municipal administrators appointed REC members, such members had “state civil servant responsibility”. The central committee was appointed by the health ministry. In England, RECs were accountable to the control body (NRES). In Canada, RECs were appointed by and reported to the hospital board. In the USA, RECs were accountable to the hospital/institution in which they were located. However, REC operational procedures were specified when registering with the control body (OHRP). Private RECs were accountable to themselves only, but their operational procedures too were specified upon registration.

#### Transparency

In Finland, REC work was not transparent (Table 4). Member selection was not an open process, there were no public annual reports of REC work and the meeting notes were available only upon request based on good grounds (as judged by the REC itself). The style of meeting notes varied. Some even used codes for projects, without mentioning the research objectives or topic and using standard phrases when giving reasons for decisions. Application documents were secret.

In England, transparency was an accepted policy and REC positions were advertised. Much information on RECs and their work, including most accepted study protocols, were available from the NRES web pages. Further openness was planned, including information on rejected projects. In Canada, REC handling notes and project protocols were kept secret. This had been identified as a transparency problem, but not solved by 2011. In the USA, REC meeting minutes were not public in most states and there was no broad discussion on lack of transparency. There was a proposal that access to research protocols be required upon trial registration; this had not occurred by 2011.

#### Obligatory education of REC members

In England, NRES provided obligatory introductory training for new REC members and old members were offered voluntary continuing training. In the other countries, REC members were not required training in ethics or in work practices. In Canada and the USA, many self-driven training options were available. In Canada, the voluntary organization for REC members and staff (CAREC) functioned as a clearing house, as well as a training and networking organizer. In the USA, the principal investigators were required to take ethics courses.

#### Variability in decisions

Variability in REC work and decisions was not discussed in Finland (Table 4). In England, this had been an important argument for streamlining the ethics system and creating the REC control body. In Canada and the USA, the issue had been recognized as a problem, but not solved. In Canada, in response, the public funder had issued detailed criteria for use in evaluations. In the USA, the problem had led to a proposal to modify the Common Rule.

#### Dealing with exceptions

##### Informed consent exemption in trials

In each country, the main mechanism for assuring voluntary participation was obtaining permission from participants fully informed of the trial (informed consent). In certain research designs, however, this did not work. In Finland, exemptions from informed consent (waivers) were not possible for medical intervention studies (Table 4); practices varied in the case of surveys and document-based studies. Researchers solved the problem of lack of exemptions by using apparent consent, changing the design, redefining the project as non-medical research or development work, or dropping it. In the other countries, RECs could grant exemptions to informed consent. In England, some interviewees stated that informed consent had become less problematic.

#### Emergency drug trials

In Finland, the EU clinical trials directive had been interpreted as requiring informed consent from patients before recruitment for emergency drug trials (Table 4). In England, although also an EU country, interpretation of the directive had changed over time and emergency drug trials were possible. This change was said to have been facilitated by lobbying by emergency care physicians. In Canada and the USA, exemptions to informed consent were used in emergency trials.

#### Quality assurance research

Research evaluating how well healthcare services or patient care works or should work (quality improvement research, quality assurance, audit, quality evaluation) was problematic for RECs in all countries (Table 4). In Finland, there had been no public discussion of the issue. RECs emphasized separating research from care; once the project had been defined as medical research, no distinction was made in its handling. In England, some advocated the exemption of quality assurance studies from the ethics approval requirement. This was part of a trend of focusing the NHS REC system on clinical patient level research and leaving health services and social sciences research outside their mandate. In Canada and the USA, practices varied – some RECs exempted such research, some had an expedited procedure with simpler requirements, and some treated them as normal research projects with full handling procedures.

## Discussion

### Study strengths and weaknesses

Most data from the four countries was collected by one person with prior knowledge of the topic, enabling the consistent external examination of each system [4]. This also enabled information from one interview to be used in the next.

Weaknesses included the difficulties inherent in any country comparison, such as those resulting from varying healthcare systems and the same tasks being handled by different actors in different countries. Aspects covered varied between interviews. Due to limited resources, it was not possible to approach informants again to enquire about issues which had arisen after their interviews. The findings presented are simplifications and do not describe the many exceptions and nuances. Data confidentiality, data access, secrecy, and privacy issues were not systematically studied, although these are topical in research ethics.

Similar previous comparative studies were not found in order to compare the results. This may be due to the fact that this study was the first of its kind, or that previous studies have appeared in forms difficult to trace via literature searches. Previous literature was unsystematically searched from medical databases using various keywords and from the reference lists of related articles.

### Comments on results

In Finland, RECs were regulated by a detailed medical research law and in case of drug trials, as in England, by a detailed EU law (directive). Detailed legislation can lead to conflicts between laws and ethics [2]: an important ethical question concerns the amount of discretion left to researchers. A check-box mentality may make them feel that they have lost responsibility for their own work. There is no one way of performing clinical research ethically; overly detailed advice will harm both research and ethics [17].

Furthermore, laws make the ethics system rigid, particularly supra-national laws. They are more difficult to change than rules in response to regulatory problems or changes in research. Broad-based legislation and detailed rules at lower level, as in the USA, may be a good compromise, allowing a more responsive system. Another legal issue concerns how much consideration RECs should give to the consequences of their decisions. In Finland, little consideration was given to this, but it was a very prominent issue in the USA: RECs have functioned as legal insurance for hospitals in regard to research.

In Finland, a key issue was that regulation was legally required for “medical” research only. This distinction from the rest of “health” research or other human research was largely artificial. In the other countries, the definitional problems were smaller, being more related to the location of the research. None of the countries had resolved how to distinguish research from other activities; the boundaries with research and public health interventions or quality assurance programs were unclear. Such problems are likely to become more prominent alongside new trends in evidence-based healthcare [2]. Such distinctions were especially difficult in Finland, whose research legislation did not allow exemptions to informed consent. Healthcare ethics should be considered as a whole rather than divided between research and healthcare practice [18,19].

In all countries, drug trials were subject to special regulation and additional reviews by drug control authorities. The special regulation system for drug trials could be questioned, but this had not been done. The 2001 EU clinical trials directive on drug research was very detailed. In 2017, it will be replaced by a new revised EU-regulation (No 536/2014 on clinical trials on medicinal products for human use), equally detailed, but improved in some respects. In the USA, drug trials aiming at drug licensing had their own regulation path, since most were handled in private RECs, with the drug authority (FDA) playing a key role. Furthermore, in the two EU countries studied, Finland and England, the directive changed some features of the overall research regulation system and rules. In general, the EU directive had been important in the creation and formulation of RECs in many European Union countries [1,7].

Regulation costs for researchers and institutions have been discussed in the USA [12,18]. Studies show that RECs imposed notable costs on host institutions [18,20,21]. However, the current study suggests that institutions did not consider REC costs too high, as RECs were an important part of risk management. In addition, there were costs for other actors, particularly researchers and sponsors.

It is not clear what constitutes the ethics to be guaranteed by RECs [6,22-24]. In addition to patient protection, the RECs in the four countries studied had other criteria of varying importance. One of them, scientific quality, can be problematic, as REC members are chosen to review participant protection and may not be the best persons to evaluate scientific quality, particularly that of studies using novel approaches. The English approach, requesting evidence of scientific value for the REC’s consideration, was a good compromise.

A lack of ethics criteria also meant that research motives were not considered. Availability of results is an ethical issue because, if the results are not publicly available, altruistic participation or the use of publicly collected information become private property.

Studies suggest that many academic researchers (i.e. those not working for commercial sponsors) were unhappy with RECs. Some have complained that RECs impede their research work rather than ensuring ethical conduct [25-31]. In this study, the quality of REC work was not directly measured, but structures or procedures for ensuring quality were studied. Finland had the least and England the most such structures. In England, researchers’ ability to choose their REC was a key issue. As RECs were not paid for their work, there was no incentive to “sell permissions”, which was also likely to be prevented by the close surveillance of the REC control body. In the USA “selling permissions” was a clear danger in the case of private RECs. Other important elements of quality assurance were transparency and appeal possibilities; these too were best organized in England. However, some appeal possibilities may be notional only, due to researchers’ fear of the negative consequences of appeals. Fearing the possible consequences, researchers seldom wish to protest openly or challenge RECs. In the case of drug trials, researchers may be afraid to challenge the control authorities for fear of repercussions when applying for a product license or another trial.

The general aim of RECs, to help preserve research ethics, is rarely questioned, but various aspects of REC activities have been widely criticized [16,24,26,32-38]. In all countries, a great deal of time was used by RECs and other actors to check various procedural formalities. Such efforts are unlikely to have made research more ethical. They may serve other purposes, but these were not clearly spelled out. Multi-location projects were increasingly common, but the REC system had been created at a time when they were less so.

This study provides no direct answers to the obvious question – why had the various identified problems not been solved. Power relations may be important: who gets to define what is right and wrong [39] and what should be done and by whom? RECs are a channel through which outsiders can participate in decision making in a research project. Furthermore, much clinical research is supported by influential technology players such as multinational drug firms, which may be influencing the content of ethics and administrative rules to protect them from competitors and academic investigators, and may also want uniform regulations and requirements for large markets. Those responsible for REC work may feel the need to resist commercial pressures and create various rules to assist in this.

## Conclusions

This study lends support to the criticism that RECs may prevent research: the rules and procedures are many and costly, and likely to deter individual clinicians [40]. General observations suggest that RECs have improved the rights of people participating in research, but the balance struck between the rights of people and patients in general is questionable if relevant research has been prevented or the relevance of research design and content compromised. The rules and practices of RECs should be improved.

Certain features of REC work in individual countries could serve as a model for others. Streamlining of the ethics committee system in England, content advice on the handling of ethical issues in Canada, and the separation of drug trials for licensing from other drug research in the USA, are examples. In regard to structure, some Finnish features, such as area responsibility, lack of private RECs, and the relative lightness of REC work, were good [41].

## Additional file

Additional file 1: Appendix. Laws, rules and guidelines, which RECs should or could use to guide their work. (PDF 79 kb)

## Abbreviations

COI: Conflicts of interest
NHS: National Health Service
NRES: National Research Ethics Service
OHRP: Office of Human Research Protection
RECs: Research ethics committees
USA: United States of America
