# Supplementary material for: Research ethics committees in the regulation of clinical research: comparison of Finland to England, Canada, and the United States
Source: Health Res Policy Syst. 2016 Jan 19;14:5. doi: 10.1186/s12961-016-0078-3 (PMC4750216; doi:10.1186/s12961-016-0078-3)
Supplement: Additional file 1: Appendix. — Laws, rules and guidelines, which RECs should or could use to guide their work. (PDF 79 kb) [file 12961_2016_78_MOESM1_ESM.pdf]

Appendix. Laws, rules and guidelines, which RECs should or could use to guide their work.

In Finland, research ethics committees had been regulated since 1999 by a medical research law and its sub-laws, specifying REC mandates and tasks. Each hospital district (each of the five university hospital districts since 2010) should establish an official REC, dealing with "medical" research projects as defined by law. In addition, several other laws relate to certain aspects of medical research, including the Patient Status and Rights Act.

In the other countries, RECs were less directly law based, but referred to by various laws. In England, RECs were specified by health ministry rules, including those of the REC control body (NRES National Research Ethics Service). Although RECs were not legal bodies in general, they were in regard to drug and device trials and some special classes, such as research with persons lacking the capacity to consent and exposure to ionizing radiation, as stipulated in specific laws.

In Canada, RECs were formalized by the requirements and guidelines of the main public funder, the Canadian Institutes of Health Research (CIHR), part of the Tri-Council funding agency. The guidelines (Tri-Council Policy Statement: Ethical Conduct for Research Involving Humans) detailed REC duties and issued stipulations on handling applications. Establishing RECs and following advice was necessary for hospitals seeking federal research money. In the USA, the research law (1974 National Research Act) mentioned the need for REC review and informed consent. REC tasks were expanded in government (federal) regulations (Common rule). The Common rule concerned all human research, but was adaptable to the research type and purpose.

In each country, laws on data confidentiality (privacy laws/freedom of information laws) existed and were integral to research regulation. I did not collect detailed information on these intricate regulations, and the role of the RECs overseeing them varied.

Research ethics codes, such as the Declaration of Helsinki [1] were not central to discussions on the REC system or work. However, codes had had an impact on national guideline formulation. In England, I received various critical comments on the content of the Helsinki Declaration and Good Clinical Practice (GCP) guidelines. Respondents indicated that the GCP guidelines were drawn up for new drug trials; commercial sponsors were happy with them, but academics researching drugs already on the market were not.

Special laws governed drug trials in all of the countries. In Finland, the extra requirements of the 2001 EU clinical trials directive formed part of the medical research law and drug law; in England they were included in the drug law. In Finland, as well as an REC review, drug trials had been surveyed by the drug control authority before the directive, but in England the drug control authority had reviewed only the drug itself; the directive had introduced a second review. Some of the directive's features had become part of general health research review principles. In England, some interviewees said that REC work was dominated by the evaluation criteria of drug trials. Some interviewees in Europe and the USA stated bluntly that the directive had failed. In England, a lively discussion was underway on how to change the directive or its interpretation in the revision of the directive (accepted in 2014 and expected to become into force in 2017, EU-regulation No 536/2014 on clinical trials on medicinal products for human use).

In Canada and the USA, the drug law covered only trials with unlicensed (new) drugs and new indications intended for use in sales licensing. In the USA, the drug law was specific in regard to

trial content, but the content was very close to the Common rule. The drug control authority (Food and Drug Administration FDA) had been proactive in setting standards and informing and educating researchers and sponsors of the related principles. In Canada, the rules of the drug control authority (Health Canada) were not specific, and the tri-council advice also applied to drug trials.
